# CT-guided Thermal Ablation of Liver Tumors Using Intraprocedural CT-CT Fusion for Applicator Position and Ablation Completeness Assessment: a Single-Center Comparative Analysis

**DOI:** 10.1007/s00270-025-04111-w

**Published:** 2025-07-10

**Authors:** Koen H. M. Verdonschot, Sjoerd F. M. Jenniskens, Peter B. van den Boezem, Eric T. T. L. Tjwa, Johannes H. W. de Wilt, Jurgen J. Fütterer, Martijn W. J. Stommel, Christiaan G. Overduin

**Affiliations:** 1 Department of Medical Imaging, Radboud University Medical Center, Nijmegen, The Netherlands; 2 Department of Gastroenterology and Hepatology, Radboud University Medical Center, Nijmegen, The Netherlands; 3 Department of Surgery, Radboud University Medical Center, Nijmegen, The Netherlands

**Keywords:** Liver malignancies, Thermal ablation, Local tumor progression-free survival (LTPFS), Image fusion

## Abstract

**Purpose:**

To compare outcomes of CT-guided thermal ablation of liver tumors with versus without use of intraprocedural CT-CT image fusion.

**Materials and Methods:**

This retrospective cohort study included all patients treated with CT-guided percutaneous thermal ablation for hepatocellular carcinoma (HCC) or colorectal liver metastases (CRLM) between January 2017 and April 2023 at our institution. From October 2019, intraprocedural CT-CT deformable image fusion (IF) using dedicated software (Vitrea, Canon Medical) was introduced to the thermal ablation procedure workflow to visually assess applicator placement before ablation and ablation completeness posttreatment. Local tumor progression (LTP) was assessed on follow-up imaging. LTP-free survival (LTPFS) between groups with and without IF was estimated with the Kaplan–Meier method and risk factors for LTP were identified with Cox regression analysis.

**Results:**

A total of 113 patients treated in 139 sessions were included; 66 treatments for 86 tumors without use of IF (56 HCC; 30 CRLM) and 73 treatments for 92 tumors with use of IF (46 HCC; 46 CRLM). Two-year LTPFS was significantly improved with use of IF for both HCC (97% vs. 74%; *p* = .009) and CRLM (82% vs. 56%; *p* = .033). On univariate regression analysis, use of IF was a predominant factor significantly associated with improved LTPFS in patients with HCC (HR: 0.21, *p* = .037) and CRLM (HR: 0.38, *p* = .042).

**Conclusion:**

In this single-center study, the use of software-based intraprocedural CT-CT image fusion for applicator position and ablation completeness assessment was associated with improved local tumor progression-free survival after CT-guided thermal ablation of HCC and CRLM.

*Level of evidence*: 3.

**Graphical abstract:**

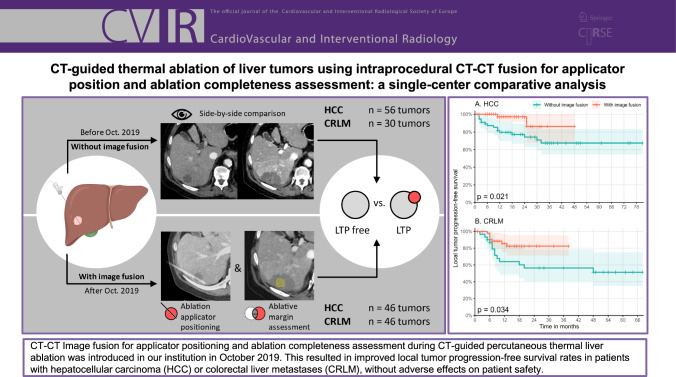

**Supplementary Information:**

The online version contains supplementary material available at 10.1007/s00270-025-04111-w.

## Introduction

Percutaneous thermal ablation, encompassing RFA and MWA, is a recognized treatment option for early-stage primary or small-size oligometastatic liver tumors. Multiple reports have shown good overall survival (OS) after thermal ablation of hepatocellular carcinoma (HCC) (3-yr: 67–75%) and colorectal liver metastases (CRLM) (3-yr: 60–78%) [1–4]. However, percutaneous thermal ablation has been associated with local tumor progression (LTP) rates on the order of 5.7–21.7% [5–7], ranging up to 50% in some smaller studies [8].

Accumulating evidence has shown that the minimal ablative margin (MAM) is one of the main predictive factors of LTP [9–12]. The current expert opinion is to completely ablate the tumor including a circumferential ablative margin of at least 5mm around the lesion [13]. However, a well-validated, standardized method for intraprocedural ablative margin assessment in clinical routine is currently lacking. The current intraprocedural assessment of ablation completeness is typically based on side-by-side comparison of pre- and immediate post-ablation contrast-enhanced CT (CECT) imaging; however, this method has been demonstrated inaccurate for determining the ablative margin in numerous studies [14–19].

Laimer et al. indicated rigid image fusion of intraprocedural acquired pre- and post-ablation CECT imaging to allow for improved assessment of ablation completeness, with the retrospectively derived quantitative ablative margin being an independent predictor of LTP [20]. Although multiple studies have reported on approaches to quantify the ablative margin, these have often been restricted to nonclinical retrospective use [21]. In recent years, medical image fusion software applications have become increasingly available, either as part of radiology software packages or integrated into scanner software, that enable co-registration and fusion overlay of intraprocedurally acquired CT images at multiple timepoints during the thermal ablation procedure. These could therefore enable a nonquantitative visual assessment of applicator position before ablation and of ablation completeness immediately post-ablation. By providing the treating physician an improved assessment of applicator position and ablation completeness as compared to traditional side-by-side comparison, this could potentially translate into improved outcomes. The use of CT-CT image fusion for applicator position and ablation completeness assessment was introduced to the clinical workflow of percutaneous CT-guided thermal ablations in our institution in October 2019.

The purpose of this study was to compare outcomes of CT-guided percutaneous thermal ablation of liver tumors with versus without use of intraprocedural CT-CT image fusion in patients with hepatocellular carcinoma or colorectal liver metastases.

## Material and Methods

### Patient Selection

In this retrospective, single-center cohort study, all patients who underwent CT-guided percutaneous thermal ablation for HCC or CRLM between January 2017 and April 2023 at our institution were included. Patients were excluded if no follow-up imaging was available. Patients were stratified into two cohorts: patients treated without the use of intraprocedural CT-CT image fusion (IF) or patients treated with the use of intraprocedural CT-CT IF during the ablation procedure. The local Institutional Review Board approved this study and waived the requirement for informed consent for retrospective study participation (CMO2018-5023).

### Thermal Ablation Procedures

All patients were discussed in a multidisciplinary tumor board meeting before percutaneous thermal ablation. The diagnosis was based on abdominal radiological imaging, by either contrast-enhanced CT or MRI consistent with HCC or CRLM. In selected cases of diagnostic uncertainty, targeted biopsy was performed to obtain histological verification.

All ablation procedures were performed in the CT suite by the same three (JJ, SJ, MA) interventional radiologists with > 10 years of experience in tumor ablation. Intraprocedural CT imaging was performed on a 128 or 320-slice CT scanner with a slice thickness of 0.5–1.0mm (Aquilion One, Canon Medical Systems). Ablations were performed under general anesthesia and controlled apnea by disconnection of the endotracheal tube was applied during al CT imaging. Patients were treated with either RFA or MWA, based on historic availability and physicians' choice depending on tumor size and location. RFA was performed with Starburst (AngioDynamics) or CoolTip (Covidien) systems. MWA was performed with Solero (AngioDynamics), NeuWave (Johnson & Johnson), or Emprint (Medtronic) systems. Ablation power and duration were determined based on tumor size and manufacturer specifications. If indicated, pneumo- or hydrodissection was used to mitigate the risk of unintended thermal damage to adjacent structures. Technical success was defined as achievement of complete tumor coverage as assessed on immediate post-ablation CECT by the treating physician [22]. 

All patients underwent imaging follow-up consisting of CECT or CE-MRI imaging every 3 months for the first year, followed by 3–6-month intervals thereafter. All follow-up imaging was reviewed by a board-certified abdominal radiologist.

### Intraprocedural Assessment of Applicator Position and Ablation Completeness

Up to October 2019, identification of the target tumor at the start of the ablation procedure was performed using ultrasound or, if not feasible, intraprocedural CECT. Ablation applicators were placed under ultrasound or CT guidance and its position was assessed with ultrasound or non-enhanced CT. CECT imaging was performed immediately post-ablation to assess the treatment result and potential complications. Ablation completeness was assessed by visual two-dimensional (2D) side-by-side comparison of pre-procedural or intraprocedural contrast-enhanced pre-ablation imaging with intraprocedural post-ablation CECT. If incomplete tumor coverage was suspected, immediate re-ablation was performed at the discretion of the treating physician.

**Fig. 1:**
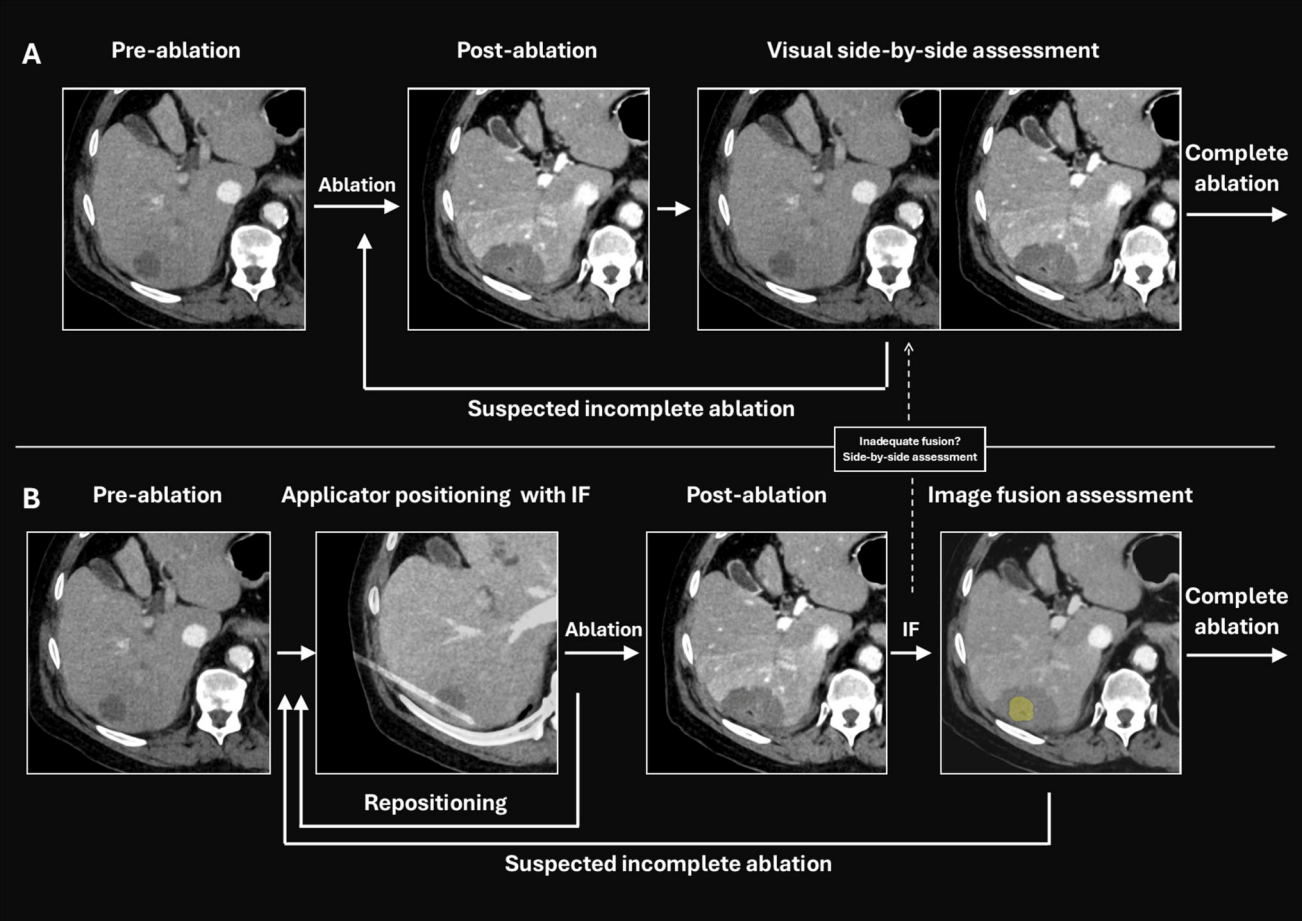
The clinical workflow for percutaneous thermal ablation: (**A**) as used before October 2019, using visual side-by-side image comparison to assess the completeness of the ablation, and (**B**) from October 2019, using image fusion to verify ablation applicator position prior to ablation and to assess ablation completeness immediately after the ablation

From October 2019 onward, identification of the target tumor at the start of the ablation procedure was performed by intraprocedural multiphase CECT. Ablation applicators were placed under ultrasound or CT guidance and its final position documented using a non-enhanced CT scan. Applicator positioning was then assessed by use of CT-CT deformable image fusion, wherein the non-enhanced CT scan showing the final applicator position was overlaid on the intraprocedural pre-ablation contrast-enhanced CT scan showing the target tumor, using dedicated image fusion software (Vitrea CT liver analysis, Canon Medical). The deformable image registration algorithm allows for correction of local tissue deformation, for example, due to breathing or post-ablation tissue shrinkage, and is specifically intended for liver co-registration in contrast-enhanced CT scans [23]. In case of inadequate applicator position, repositioning or ablation in additional positions could be performed at the discretion of the treating physician. Immediately post-ablation, a multiphase CECT scan was performed to assess treatment results and potential complications with the use of deformable CT-CT image fusion of the intraprocedural pre-ablation CECT scan showing the target tumor and the intraprocedural immediate post-ablation CECT showing the ablation zone using the same software. Image fusion quality was assessed by the treating physician on the alignment of local intrahepatic landmarks, predominantly liver vessels, using a slider to control transparency of the superimposed pre- and post-ablation CECT in the overlay view. Upon confirmation of adequate image fusion quality, ablation completeness was assessed in the overlay view by visual inspection of tumor and ablation margin coverage in multiplanar views. If image fusion quality was deemed inadequate, defines as gross mismatch between local landmarks and liver contour, conventional side-by-side comparison was used to assess ablation completeness. If incomplete coverage was suspected, immediate re-ablation was performed at the discretion of the treating physician. The two workflows are schematically depicted in Fig. 1.

### Outcome Definitions

Ablation outcomes were assessed following the standardized reporting guidelines in image-guided tumor ablation [22, 24]. Local tumor progression was defined as a new tumor focus within or directly adjacent to the ablation zone on contrast-enhanced follow-up imaging after intraprocedural post-ablation imaging documented no evidence of residual tumor. Time to local tumor progression was defined as the time between thermal ablation and detection of LTP. In patients who underwent a liver transplantation, the date of last follow-up imaging was noted or the date of transplantation if subsequent histopathological analysis was available and showed no local tumor progression.

Perivascular and subcapsular tumor locations were defined as tumors within close proximity (< 5mm) of a major hepatic vessel or the liver capsule, respectively. An ablation position was defined as each unique position where an ablation was performed, using either a single applicator or multiple applicators. Immediate re-ablations were defined as additional ablations performed in the same treatment session following ablation completeness assessment with CECT imaging. Complications following the procedure were recorded according to the Common Terminology Criteria for Adverse Events (CTCAE, version 5.0). All subsequent treatments (i.e., locoregional or systemic) given after the thermal ablation procedure were recorded and for locoregional treatments were classified as related to the initial target lesion of the ablation procedure (i.e., where local tumor progression of the initial target lesion was or contributed to the indication for additional locoregional treatment).

### Statistical Analysis

Results were assessed separately for HCC and CRLM and compared between the cohort treated with use of IF and without use of IF. LTPFS was analyzed on a per-tumor basis with the Kaplan–Meier method. Survival curves between groups were compared with the log-rank test. Association of use of IF and other variables with LTPFS was assessed using Cox regression analysis. Continuous patient and tumor characteristics between subgroups were tested with Student’s t-test or Mann–Whitney U-test, based on normality distribution determined by the Shapiro–Wilk test. Categorical characteristics were compared with Fisher’s exact test. Data are expressed as mean with standard deviation or median with interquartile range (IQR) as appropriate. All statistical analyses have been conducted using R and RStudio (Posit Software, http://www.posit.co). P-values < 0.05 were considered statistically significant.

## Results

### Patient Cohort

**Fig. 2:**
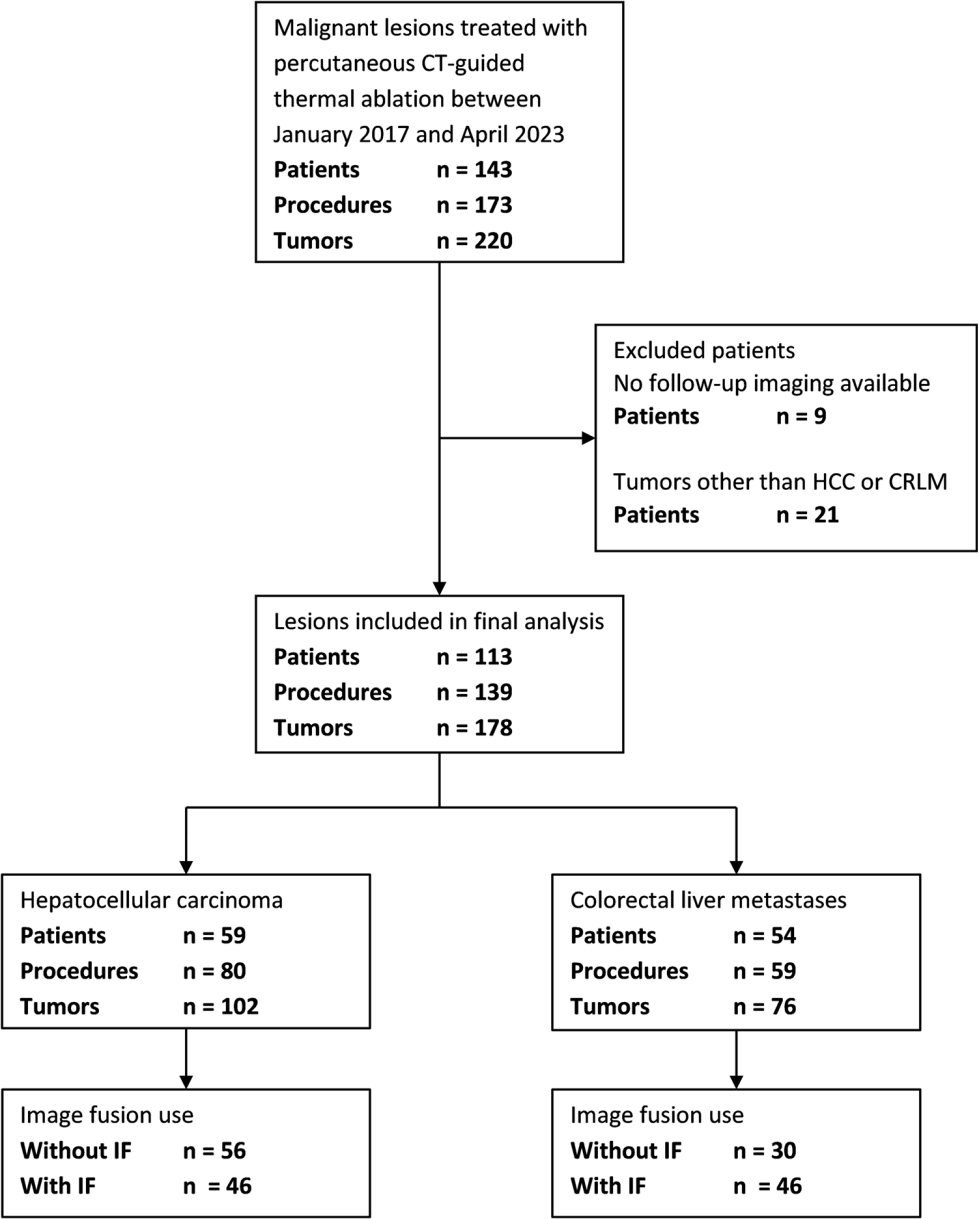
Flowchart of in- and exclusion criteria for final analysis

Between January 2017 and April 2023, a total of 143 patients were treated with CT-guided percutaneous thermal ablation in 173 procedures for 222 malignant liver tumors. Thirty patients were excluded due to absence of follow-up imaging (*n* = 9) or histotype other than HCC or CRLM (*n* = 21) Therefore, a total of 113 patients treated in 139 procedures for 178 tumors were included for final analysis; 66 procedures for 86 tumors without use of IF and 73 procedures for 92 tumors with use of IF (Fig. 2).

### Hepatocellular Carcinoma

Fifty-nine patients were treated for a total of 102 hepatocellular carcinomas, of which 56 tumors (55%) in 41 procedures without IF versus 46 tumors (45%) in 39 procedures with IF. In 5 patients, treated for 9 tumors, IF was available but could not be used due to technical issues or absence of application specialist. Baseline patient, tumor, and treatment characteristics are presented in Table 1. Ablation modality (*p* = 0.004), number of ablation probes (*p* = 0.004), and the number of ablation positions per tumor (*p* < 0.001) were significantly different between both treatment groups. Sixteen immediate re-ablations were performed after ablation completeness assessment; in 7/56 (13%) tumors in the group without IF, compared to 9/46 (20%) tumors in the group with IF (*p* = 0.415). Complication rates did not differ between subgroups; 7/41 (17%) without IF versus 8/39 (21%) with IF (*p* = 0.779).

**Table 1:** Baseline patient, tumor, and treatment characteristics for patients with hepatocellular carcinoma (HCC), treated with percutaneous thermal ablation. Statistically significant differences are marked in bold. IQR = Interquartile range

Patient, tumor, and treatment characteristics, HCC		Without IF (%)		With IF (%)		*p*-value
*Patient characteristics*		*n* = 41		*n* = 39		
Gender	Female	14	(34)	13	(33)	1.000
	Male	27	(66)	26	(67)	
Age (median (IQR))		71 (64–76)		70 (64–75)		.494
Child–Pugh classification	A	28	(50)	37	(8)	.075
	B	7	(13)	2	(4)	
	Unknown	21	(37)	7	(15)	
*Tumor characteristics*		*n* = 56		*n* = 46		
Tumor size, mm (median (IQR))		18 (14–24)		15 (12–20)		.178
Tumor size	1-10mm	7	(13)	4	(9)	.207
	11-20mm	31	(55)	32	(69)	
	21-30mm	11	(19)	9	(20)	
	> 30mm	7	(13)	1	(2)	
Perivascular location	Yes	5	(9)	8	(17)	.242
	No	51	(91)	38	(83)	
Subcapsular location	Yes	26	(46)	16	(34)	.312
	No	30	(54)	30	(66)	
De Novo	Yes	51	(91)	38	(83)	.242
	No	5	(9)	8	(17)	
*Treatment characteristics per procedure*		*n* = 41		*n* = 39		
No. of treated tumors in session	1	29	(71)	31	(79)	.709
	2	11	(29)	7	(18)	
	3	1	(2)	1	(3)	
Complications	Yes	7	(17)	8	(21)	.779
	No	34	(83)	31	(79)	
*Treatment characteristics per tumor*		*n* = 56		*n* = 46		
Technical success		56	(100)	46	(100)	1.000
Ablation Modality	RFA	9	(16)	0	(0)	**.004**
	MWA	47	(84)	46	(100)	
Image guidance for applicator placement	US	17	(30)	9	(20)	.258
	CT	39	(70)	37	(80)	
No. of ablation applicators	1	47	(83)	30	(65)	**.042**
	2	7	(13)	15	(33)	
	3	2	(4)	1	(2)	
No. of ablation positions	1	48	(86)	22	(47)	** < .001**
	2	7	(12)	23	(50)	
	≥ 3	1	(2)	1	(2)	
Immediate intraprocedural re-ablation	Yes	7	(14)	9	(20)	.415
	No	49	(86)	37	(80)	

**Fig. 3:**
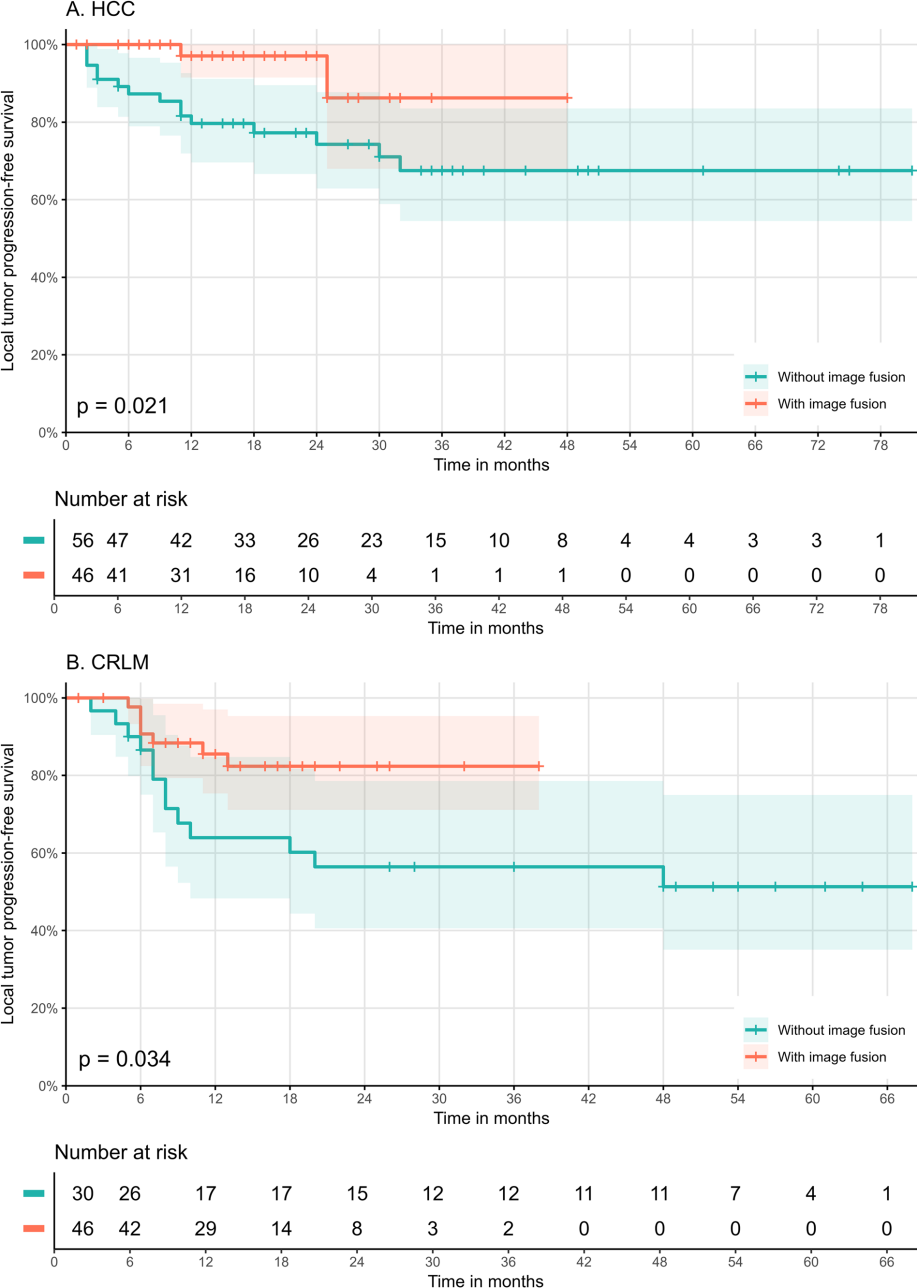
Kaplan Meier graph for local tumor progression free survival for (**A**) hepatocellular carcinoma (HCC), and (**B**) colorectal liver metastases (CRLM) treated without and with use of image fusion assessment

Median follow-up time was 21 months (range: 2–81 months) versus 15 months (range: 1–48 months) between subgroups without and with IF. Overall, 17 tumors developed LTP after a median time to progression of 11 months (range: 2–32 months). Cumulative LTP rates were 15/56 (27%) for the group without IF and 2/46 (4%) for the group with IF (*p* = 0.014). Median time to progression was 9 months (range: 2–32 months) in the group without IF and 18 months (range: 11–25 months) with IF and did not differ significantly between both groups (*p* = 0.330). One-year (79% vs. 97%) and two-year (74% vs. 97%) LTPFS rates were significantly improved between tumors treated without IF versus with IF (*p* = 0.009), as shown in Fig. 3.

Univariate Cox regression analysis revealed image fusion as the only factor significantly associated with LTPFS (HR: 0.21, *p* = 0.037), as shown in Table 3.

### Colorectal Liver Metastases

Fifty-four patients were treated for a total of 76 colorectal liver metastases, of which 30 tumors (39%) in 25 procedures without IF versus 46 tumors (61%) in 34 procedures with IF. Baseline patient, tumor, and treatment characteristics are presented in Table 2. The ratio of de novo liver metastases (*p* = 0.012), the image guidance for applicator placement (*p* = 0.002), and the number of ablation positions per tumor (*p* < 0.001) were significantly different between both treatment groups. Overall, 10 immediate re-ablations were performed after insufficient ablation was suspected on ablation completeness assessment, with no difference between subgroups treated without versus with IF (*p* = 1.000). Complication rates did not differ between subgroups; 5/25 (20%) without IF versus 2/34 (6%) with IF (*p* = 0.122).

**Table 2:** Baseline patient, tumor, and treatment characteristics for patients with HCC, treated with percutaneous thermal ablation. Statistically significant differences (*p* < 0.05) are marked in bold. CTX = chemotherapy, IQR = interquartile range

Patient, tumor, and treatment characteristics, CRLM		Without IF (%)		With IF (%)		*p*-value
*Patient characteristics*		*n* = 25		*n* = 34		
Gender	Female	7	(28)	12 (35)	(35)	.587
	Male	18	(72)	22 (65)	(65)	
Age (median (IQR))		67 (59–75)		68 (58–77)		.494
Neo adj. CTX	Yes	10	(40)	14 (41)	(41)	1.000
	No	15	(60)	19 (59)	(59)	
*Tumor characteristics*		*n* = 30		*n* = 46		
Tumor size, mm (median (IQR))		17 (11–22)		14 (11–20)		.178
Tumor size	1-10 mm	6	(20)	11	(24)	.918
	11-20 mm	15	(50)	24	(52)	
	21-30 mm	8	(27)	9	(20)	
	> 30 mm	1	(3)	2	(4)	
Perivascular	Yes	4	(13)	13	(28)	.164
	No	26	(87)	33	(72)	
Subcapsular	Yes	12	(40)	11	(24)	.201
	No	18	(60)	35	(76)	
De Novo	Yes	29	(97)	34	(74)	**.012**
	No	1	(3)	12	(26)	
*Treatment characteristics per procedure*		*n* = 25		*n* = 34		
No. of treated tumors in session	1	21	(84)	24	(71)	.548
	2	3	(12)	8	(23)	
	3	1	(4)	2	(6)	
Complications	Yes	5	(20)	2	(6)	.122
	No	20	(80)	32	(94)	
*Treatment characteristics per tumor*		*n* = 30		*n* = 46		
Technical success		30	(100)	46	(100)	1.000
Ablation Modality	RFA	4	(13)	1	(2)	.076
	MWA	26	(87)	45	(98)	
Image guidance for applicator placement	US	11	(37)	3	(7)	**.002**
	CT	19	(63)	43	(93)	
No. of ablation applicators	1	25	(83)	29	(63)	.146
	2	4	(13)	14	(30)	
	3	1	(4)	3	(7)	
No. of ablation positions	1	24	(80)	16	(35)	** < .001**
	2	4	(13)	24	(52)	
	≥ 3	2	(7)	7	(15)	
Immediate intraprocedural re-ablation	Yes	4	(13)	6	(13)	1.000
	No	26	(87)	40	(87)	

Median follow-up time was 23 months (range: 2–68 months) versus 13 months (range: 1–38 months) between subgroups without and with IF. Overall, 20 tumors developed LTP after a median time to progression of 7 months (range: 2–48 months). Cumulative LTP rates were 13/30 (43%) for the group without IF and 7/46 (15%) for the group with IF (*p* = 0.047). Median time to progression was 8 months (range: 2–48 months) in the group without IF and 11 months (range: 8–25 months) with IF, but again did not differ significantly between both groups (*p* = 0.207). One-year (64% vs. 86%) and two-year (56% vs. 82%) LTPFS rates were significantly improved between tumors treated without versus with IF (*p* = 0.009), as shown in Fig. 3.

Univariate Cox regression revealed image fusion as a factor significantly associated with LTPFS (HR: 0.38, *p* = 0.042), Table 3. Besides image fusion, male sex (HR: 0.35, *p* = 0.022) and tumor size (HR: 1.06, *p* = 0.030) were also significantly associated with LTPFS.

**Table 3:** Univariate cox regression analysis of potential variables influencing the risk of LTP. Significant outcomes (*p* < 0.05) are marked in bold. HR = Hazard ratio, CI = confidence interval

Prognostic factors		HCC		CRLM	
		HR (95% CI)	*p*-value	HR (95% CI)	*p*-value
Age		0.98 (0.93–1.04)	.510	0.97 (0.94–1.01)	.185
Sex	female	*reference*		*reference*	
	male	1.28 (0.45–3.66)	.644	**0.35 (0.14–0.86)**	**.022**
Child–Pugh	A	*reference*		–	
	B	1.30 (0.28–6.03)	.739	–	
Tumor size		1.02 (0.97–1.0)	.506	**1.06 (1.01–1.13)**	**.030**
Neo adj. chemotherapy	no	–		*reference*	
	yes	–		0.93 (0.37–2.33)	.876
Perivascular location	no	*reference*		*reference*	
	yes	0.98 (0.22–4.30)	.979	0.37 (0.09–1.62)	.188
Subcapsular location	no	*reference*		*reference*	
	yes	1.56 (0.60–4.05)	.363	1.71 (0.70–4.19)	.243
Ablation modality	MWA	*reference*		*reference*	
	RFA	1.55 (0.44–5.47)	.494	1.47 (0.34–6.32)	.608
No. of treated tumors	solitary	*reference*		*reference*	
	multiple	1.34 (0.52–3.48)	.545	0.57 (0.22–1.48)	.249
Ablation applicators	1	*reference*		*reference*	
	2	0.84 (0.24–2.95)	.780	0.58 (0.17–2.03)	.398
	3	–	–	2.05 (0.46–9.07)	.344
Ablation positions	1	*reference*		*reference*	
	2	0.35 (0.08–1.52)	.160	0.81 (0.30–2.18)	.675
	≥ 3	–	–	0.76 (0.10–5.89)	.791
Image guidance for applicator placement	CT	*reference*		*reference*	
	US	1.02 (0.36–2.90)	.970	1.27 (0.46–3.54)	.644
Intraprocedural image fusion use	no	*reference*		*reference*	
	yes	**0.21 (0.05–0.91)**	**.037**	**0.38 (0.15–0.96)**	**.042**

## Discussion

This study aimed to evaluate CT-guided thermal ablation of liver tumors with and without use of intraprocedural CT-CT image fusion for assessing applicator position and ablation completeness within the clinical workflow. Patients treated with intraprocedural CT-CT image fusion showed a reduced LTP rate and improved LTPFS compared to those treated without the use of IF, for both HCC and CRLM. Additionally, the number of ablation positions per tumor increased in patients treated with intraprocedural IF, while safety profile between both groups was unaffected.

Previous studies describing the use of IF in liver thermal ablation were limited to either retrospective margin assessment after the ablation procedure or real-time assessment with ultrasound guidance [25], with two comparative studies reporting improved LTP rates using IF with (CE)US [26, 27]. However, CT guidance has been adopted by many institutions due to the superior spatial resolution and volumetric information, which could potentially facilitate more accurate 3D assessment of ablation completeness [10]. In addition, several methods have been proposed to improve patient outcomes after ablation, like stereotactic guidance [28], CT hepatic arteriography (CTHA) [29], or hepatic arteriography and C-Arm CT-guided ablation (HepACAGA) [30]. Notably, all of these methods incorporated image fusion to some extent to assess ablation completeness intraprocedurally. Wijnen et al. compared conventional CT-guided ablation without image fusion to the HepACAGA-technique, integrating rigid image fusion of pre- and post-ablation contrast-enhanced cone-beam CT imaging, and found a reduction in LTP comparable to our findings [30]. Laimer et al. state the use of intraprocedural image fusion to be one of the key factors for successful stereotactic radiofrequency ablation [28]. Conceptually, improved assessment of applicator position adequacy and ablation completeness is both factors contributing to achieving sufficient ablation margins. As several studies have shown the ablation margin to be the most important predictor of local tumor progression, this likely represents the mechanism contributing to reduced LTP rates across these and the current study [31, 32]. 

The intraprocedural use of image fusion to qualitatively assess ablation completeness was a significant contributor to improved local outcomes. Although qualitative assessment with CT-CT image fusion in itself has not been studied as a factor for reduced LTP rates before, numerous studies have identified (retrospectively measured) increased ablative margins to be one of the main prognostic factors for improved local control rates [9–11, 31, 33]. In our study, image fusion enabled a better assessment of the ablation completeness and ablative margins. Even though this did not lead to more immediate re-ablations being performed when compared between the two cohorts, it allowed for more accurate redirection of additional ablations, if necessary. Furthermore, the confirmation of ablation applicator positioning with IF prior to ablation led to significantly more ablations being performed in multiple positions. This allowed the operator to immediately optimize the procedure to ensure sufficiently large ablative zones and potentially increased ablation margins. Our findings are consistent with the results of the recently published COVER-ALL trial, the first randomized trial to compare visual side-by-side assessment to software-based deformable image fusion, for applicator positioning and quantitative ablation completeness assessment [34]. This trial demonstrated significantly larger minimal ablative margins and an increased number of multiple overlapping ablations in the group treated with use of software-based image fusion assessment, in agreement with our observations. 

Several differences were observed in the treatment characteristics between the two treatment groups. The differences in ablation type distribution and number of used ablation applicators reflect to a large degree the historical trend of the introduction of MWA as an alternative for RFA at our center and the introduction of a multi-probe device. Although these factors were not associated with local tumor progression on univariate analysis, any influence on local outcomes cannot be fully excluded. Several studies however have shown comparable results in terms of local control between both ablation modalities and consequently achieving sufficient ablative margins remains paramount to reducing the risk of LTP [35–37].

Our study had several limitations. Firstly, this was a retrospective, single-center study which may be prone to bias. Various patient and treatment characteristics changed over time due to the adoption of new ablative techniques. While IF was a strong factor associated with improved LTPFS, it was not possible to correct for other factors in a multivariable Cox regression analysis, due to limited number of events. Additionally, follow-up time was inherently shorter in the more recent cohort treated with IF, albeit only patients who were at least 12 months post-treatment were included. In previous studies, the majority of LTP incidence was seen in the first year post-treatment [38], nonetheless this may impact observed LTP rates. Thirdly, it was not possible to compare achieved ablative margins between both groups as the group without IF lacked standardized intraprocedural contrast-enhanced pre-ablation imaging required to perform this calculation. It was therefore not possible to assess if the use of image fusion resulted in the achievement of larger ablative margins. Lastly, the quality of image fusion is assessed qualitatively and may not be of sufficient quality in all patients due to motion or pneumo- or hydrodissection. This can lead to subjective interpretation of the ablation completeness. Further work is needed to develop reliable metrics for image fusion quality assessment and quantitative ablation confirmation to allow for fast, user-independent and validated intraoperative assessment methods of the ablative margins. Despite these limitations, this study provides comparative data on outcomes and indicates clinical benefits of using CT-CT image fusion to assess applicator position and ablation completeness during percutaneous thermal ablation. 

## Conclusion

In this single-center study, the use of intraprocedural CT-CT image fusion for applicator position and ablation completeness assessment was associated with improved local tumor progression-free survival after CT-guided thermal ablation of HCC and CRLM. Further research into quantitative, validated, and user-independent assessment of treatment success and margin status is paramount to improve local outcomes of percutaneous thermal ablation for liver tumors.

## Supplementary Information

Below is the link to the electronic supplementary material.

Supplementary file1 (DOCX 14 KB)
