# Supplementary material for: CT-guided Thermal Ablation of Liver Tumors Using Intraprocedural CT-CT Fusion for Applicator Position and Ablation Completeness Assessment: a Single-Center Comparative Analysis
Source: Cardiovasc Intervent Radiol. 2025 Jul 10;48(9):1327–38. doi: 10.1007/s00270-025-04111-w (PMC12464092; doi:10.1007/s00270-025-04111-w)
Supplement: Supplementary file 1 — Supplementary file1 (DOCX 14 KB) [file 270_2025_4111_MOESM1_ESM.docx]

# Supplementary materials

**HCC**

Fifteen patients had complications following the ablation; twelve grade 1 complications and three grade 3 complications.

The number of locoregional subsequent treatments related to the initial target lesion decreased between the group with IF (1/46, 2%) and without IF (13/56, 23%; p = 0.06). Locoregional treatments consisted of thermal ablation (n = 10), transarterial radioembolization (TARE) (n = 3), and transarterial chemoembolization (TACE) (n = 1). The number of patients undergoing subsequent treatments due to disease progression was also lower in the group with IF (13/39, 33%) versus without IF (28/41, 68%; p = 0.003). In total, thirty-four patients received 57 subsequent treatments, consisting of thermal ablation (n = 23), liver transplantation (n = 13), systemic therapy (n = 9), TARE (n = 8), TACE (n = 3), partial hepatectomy (n = 1).

**CRLM**

Seven patients had complications following the ablation; four grade 1 complications, one grade 2, one grade 3, and one grade 4 complication.

The number of locoregional subsequent treatments related to the initial target lesion trended towards a decrease between the group without IF (9/30, 3%) and with IF (6/46, 13%; p = 0.084). Locoregional treatments consisted of partial hepatectomy (n = 11), thermal ablation (n = 3), and radiotherapy (n = 1). The number of patients undergoing subsequent treatments due to disease progression remained similar between subgroup without IF (14/25, 44%) and with IF (17/34, 50%; p = 0.793). In total, thirty-one patients received 57 subsequent treatments, consisting of partial hepatectomy (n = 15), systemic therapy (n = 12), thermal ablation (n = 7), TARE (n = 1), and radiotherapy (n = 1).
